# OEIS complex

**DOI:** 10.11604/pamj.2013.16.129.3166

**Published:** 2013-12-05

**Authors:** Nadia Khoummane, Sanae Abakka

**Affiliations:** 1 Department of Gynecology and Obstetrics, Oncology and High Risk Pregnancies, Rabat, Morocco

**Keywords:** OEIS complex, omphalocele, anal imperforation, spinal defect

## Image in medicine

OEIS is a malformative complex that associates an omphalocele, an exstrophy of the cloaca, an anal imperforation and spinal defects. Normal development of the cloaca gives origin to the lower abdominal wall with bladder, intestine and anus, genitals organ and part of the pelvis bones and lumbosacral spine. Incidence of OEIS is rare, thought to occur in 1 in 200,000 to 1 in 250,000 live births. Clinical findings are numerous. Main findings are: failure of fusion of the genital tubercles and pubic rami, incomplete development of the lumbosacral vertebrae with spinal dysraphism, imperforate anus, cryptorchidism and epispadias in males and anomalies of the mullerian duct derivatives in females, and a wide range of urinary tract anomalies. Most patients have a single umbilical artery. Most cases are sporadic. The exact etiology remains unknown but teratogenic exposure (to diazepam, diphenylhydantoin), single defects in blastogenesis and early development of the mesoderm as well as mutations in Homeobox genes (HLXB9) have been suggested in playing a role in the pathogenesis of this disease. We relate the case of a newborn with no prenatal care, born at 35 weeks (no antenatal ultrasound performed). We thus emphasize the importance of screening ultrasound in order to diagnose this complex syndrome.

**Figure 1:**
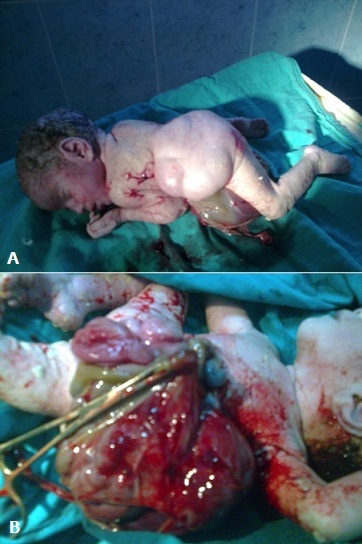
A) 35 weeks old newborn with OEIS complex. Note the anal imperforation and spinal defects; B) 35 weeks old newborn with OEIS complex. Here we can appreciate an omphalocele and an exstrophy of the cloaca

